# Crosstalk between the microbiota and intestinal γδ T cell compartments in health and IBD

**DOI:** 10.1080/19490976.2025.2604908

**Published:** 2025-12-29

**Authors:** Ananya Parthasarathy, Tingting Li, Karen L. Edelblum

**Affiliations:** a Department of Pathology, Molecular and Cell-Based Medicine and Precision Immunology Institute, Icahn School of Medicine at Mount Sinai, New York, NY, USA

**Keywords:** γδ T cell, intraepithelial lymphocyte, lamina propria, microbiota, commensal bacteria, inflammatory bowel disease

## Abstract

Unconventional T cells expressing the γδ T cell receptor (TCR) are abundant within the intestine and largely function as ‘first responders’ to injury, infection and inflammation. To this end, murine γδ T cells are highly compartmentalized within the intestinal mucosa based on the expression of their Vγ chain and their effector function. The activation status also differs among these γδ T cell populations to ensure a timely and appropriate response within their local microenvironment. In this review, we will examine the role of γδ T cell populations in the epithelium (i.e. intraepithelial lymphocytes), the lamina propria and Peyer's patches and discuss the influence of the gut microbiota on the maintenance and effector function of each compartment. We will also highlight how γδ T cells contribute to the host response to luminal bacteria and how this reciprocal crosstalk is disrupted in the context of inflammatory bowel disease (IBD). An enhanced understanding of how γδ T cells function within distinct mucosal compartments and their regulation by commensal bacteria may lead to the development of novel microbiome-based therapies for IBD.

## Introduction

The gastrointestinal tract serves as the largest internal barrier surface separating the immune system from the contents of the intestinal lumen. In addition to luminal dietary antigens and environmental toxins, the gut harbors symbiotic microbiota that includes trillions of bacteria, viruses and fungi^1^ that contribute to nutrient bioavailability and prime an appropriate response to enteric pathogens. Compared to conventional T cells, the relationship between commensals and unconventional T cells expressing the γδ T cell receptor (TCR) is relatively understudied. In this review, we will examine the compartmentalization of γδ T cells within the mucosa, how the gut microbiota influences these γδ T cell populations and their function, and how this crosstalk is disrupted in the context of inflammatory bowel disease (IBD). Further, we will discuss recent advances in the field that have uncovered novel mechanisms of regulation and functions for these ‘first responders’ at one of the largest barrier surfaces in the body.

## Gut-associated lymphoid tissue

**Figure 1.**
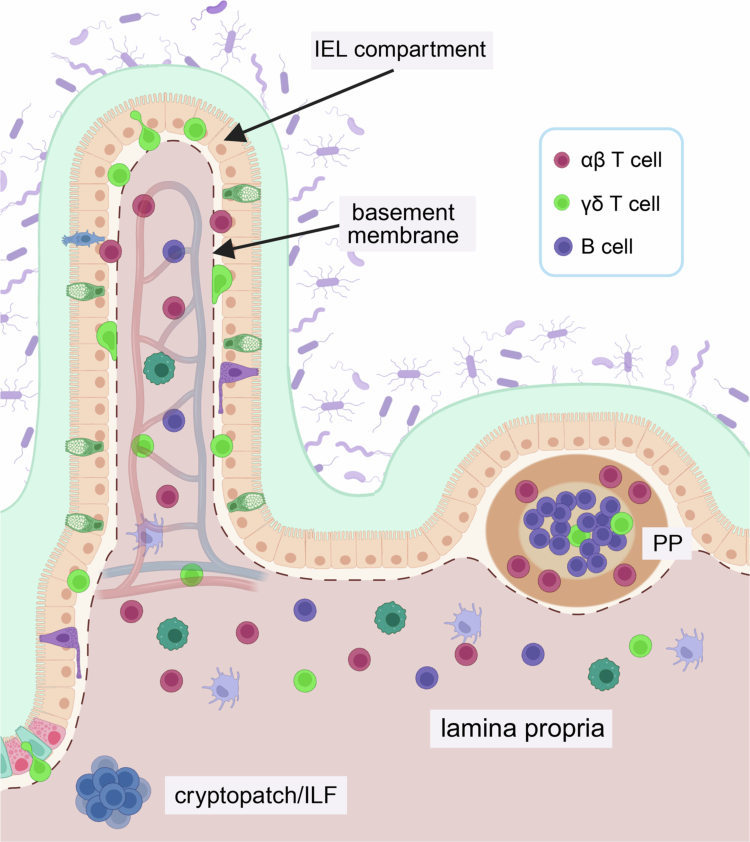
Immune induction occurs in gut-associated lymphoid tissue. The intestinal barrier is comprised of a single layer of epithelial cells separating luminal contents from the underlying lamina propria (LP). Differentiated epithelial cells arise from intestinal crypts. Within the epithelium, intraepithelial lymphocytes (IEL) are a subset of T cells that are located along the basement membrane and between adjacent enterocytes. In contrast, the LP contains a wider array of immune cells, including B cells, T cells, innate lymphoid cells (ILC), macrophages and dendritic cells. Cryptopatches and isolated lymphoid follicles (ILF) are smaller lymphoid aggregates located within the mucosa, whereas Peyer's patches (PP) contain germinal centers covered by a follicle-associated epithelium. Created in BioRender.

The intestine is continuously exposed to external factors, including both commensal and pathogenic microorganisms; therefore, a careful balance must be struck to ensure that invading pathogens are cleared while limiting immunoreactivity to dietary antigens and the gut microbiota. Commensal bacteria play a critical role in shaping intestinal mucosal immunity, both in the formation of specialized structures collectively referred to as gut-associated lymphoid tissue (GALT) and in the development of tolerogenic responses that prevent aberrant immune activation in response to commensal antigens.^2^ GALT include the mesenteric lymph node (MLN), Peyer's patches (PP), cryptopatches and isolated lymphoid follicles (ILF),^3^^,^^4^ which are sites of immune induction (Figure 1). In the MLN, naïve lymphocytes are presented antigen leading to their activation, differentiation and egress into the intestinal mucosa.^5^ PPs are distributed along the length of the small intestine and contain germinal centers that serve as sites for the generation of commensal-specific IgA. Similarly, ILFs contain germinal centers thought to be involved in the development of IgA plasmablasts,^6^ whereas cryptopatches are small lymphoid aggregates located near crypts that harbor T cell precursors.^7^

The majority of intestinal immune cells are dispersed throughout the intestinal mucosa in one of two compartments: the epithelium or the underlying lamina propria (LP). As the name suggests, intraepithelial lymphocytes (IEL) are located along the basolateral aspect of the epithelium and between adjacent epithelial cells. IELs can be subdivided into two main groups based on their expression of CD4 and/or CD8 coreceptors.^8^ Induced IELs are conventional antigen-specific T cells expressing the αβ T cell receptor (TCRαβ) along with CD4 or CD8αβ. These lymphocytes home to the gut epithelium in response to antigen encounter in the periphery. Notably, a small population of LP CD4 Foxp3^+^ Tregs can acquire CD8αα after homing to the epithelium, leading to the development of double-positive (DP) IELs.^9-11^ In contrast, natural IELs are comprised of unconventional T cells expressing the CD8αα homodimer along with TCRγδ or TCRαβ, whose activation occurs in a MHC-independent manner. Natural IELs dominate the IEL compartment, with γδ T cells accounting for nearly 60% of the IEL population in mice. While most T cells in the LP are conventional CD4 and CD8 T cells, a small population of γδ T cells is also found here.

At first glance, it might appear that categorizing γδ T cells by their location within the mucosa purely reflects a spatial classification. However, it is thought that γδ T cells have adapted to their compartment, with each population displaying unique characteristics associated with the regulation of their effector function. This adaptation is particularly important in the context of how γδ T cells contribute to the host response to luminal bacteria. γδ IELs reside within the epithelium in close proximity to commensals, whereas γδ lamina propria lymphocytes (LPL) are less likely to directly encounter commensal antigens under steady-state conditions. In the context of infection, γδ T cells in both compartments contribute to host defense through distinct mechanisms. This spatial segregation inevitably influences the cellular neighborhood surrounding γδ T cells in terms of cellular interactions and communication. γδ IELs interact primarily with intestinal epithelial cells, whereas γδ LPLs encounter a broader range of immune cells. Thus, it would be expected that the signals disseminating from gut microbes or ‘input’ to γδ T cells within each compartment would be quite different, and likewise, the ‘output’ of that information is unique to γδ IELs or γδ LPLs. Moreover, γδ T cells in PPs have their own unique phenotype.^12^ While we have emphasized the importance of γδ T cell location within the mucosa, the composition of the gut microbiome varies across the length of the gut, as does the distribution of γδ T cells. In mice, γδ IELs are more prevalent in duodenum with their number decreasing down toward the distal colon;^13^ thus, there is an inverse correlation between γδ IEL number and microbial diversity and density. The regional characterization of γδ LPLs is less thoroughly characterized, perhaps because of their relative scarcity under homeostatic conditions. Throughout this review, we will highlight the functional characteristics of γδ T cells within each compartment and the reciprocal interactions between these sentinel lymphocytes and the microbiota.

## Ontogeny and homing of intestinal γδ T cells

Specification of γδ T cell fate and compartmentalization starts early in development. Thymic development of γδ T cells is induced by a strong TCR signal, while a weak TCR signal induces TCRαβ development.^14^^,^^15^ Moreover, the development of γδ T cells occurs in waves throughout embryogenesis and into the postnatal period.^16^ In mice, each wave reflects the development of a different subset of γδ T cells defined by the Vγ chain of the TCR, which we will refer to using the Heilig and Tonegawa nomenclature.^17^ The first wave consists of Vγ5 T cells that home to the epidermis,^16^ followed by Vγ6 and Vγ4 T cells, which migrate to secondary lymphoid organs and tissue compartments including the intestinal LP.^18^ Vγ7 T cells subsequently appear in the IEL compartment and arise from either the thymus or extrathymic sites.^19^ Since γδ IELs are present in euthymic, nude mice, it is thought that these unconventional IELs can develop in cryptopatches and receive maturation signals following their migration into the epithelium.^20^^,^^21^ Lastly, Vγ1 and Vγ4 T cells develop postnatally and are largely found in circulation, retaining the ability to be recruited to peripheral tissues.

**Figure 2.**
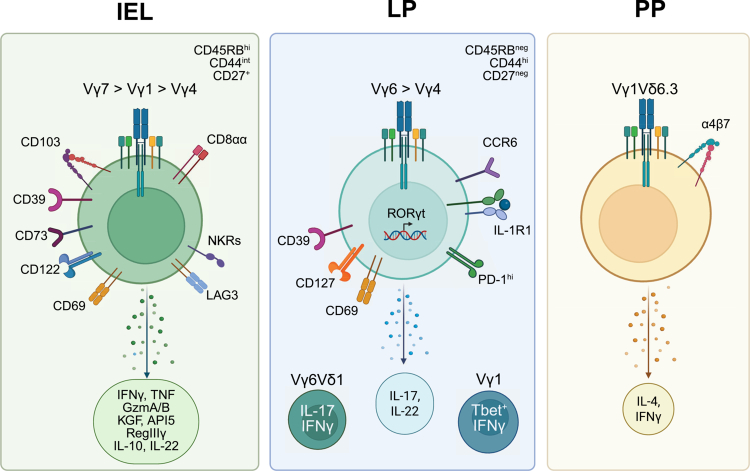
Immune profile and effector functions of intestinal γδ T cells. γδ T cells located in different intestinal immune compartments exhibit distinct cell surface receptor profiles, Vγ usage and effector functions. The IEL compartment contains γδ^IFN^ cells expressing either the Vγ7, Vγ1 or Vγ4 TCR and the CD8αα homodimer. γδ IELs express various receptors associated with tissue residency, innate immunity (i.e. natural killer receptors, NKRs) as well as inhibitory receptors to maintain a state of immunological quiescence. Further, these IELs exhibit a broad range of effector functions, including the ability to produce proinflammatory cytokines, cytolytic molecules, antimicrobial peptides and factors involved in epithelial homeostasis. γδ LPLs express the Vγ6 or Vγ4 TCR and are characterized by the production of IL-17 and IL-22, which is associated with the expression of IL-1R1, CD127, CCR6, and PD-1. In addition, Vγ1^+^ IFNγ-producing cells can be found within the LP, and polyfunctional memory Vγ6Vδ1 T cells are induced following primary infection with *Listeria monocytogenes.* γδ T cells in PP primarily express the Vγ1Vδ6.3 TCR and secrete both IL-4 and IFNγ. Created in BioRender.

The strength of TCR signaling is not only important for γδ T cell lineage commitment but also determines commitment to an effector fate.^14^^,^^22^ Those γδ T cells receiving a strong TCR signal develop into CD27^+^ Tbet^+^ IFNγ-producing γδ T cells (γδ^IFN^), whereas weaker signals lead to the differentiation of CD27^−^ RORγt^+^ IL-17a^+^ γδ T cells (γδ^17^).^14^^,^^23-26^. Metabolic programming also plays a major role in the differentiation of γδ T cell effector subsets;^27^ a strong TCR signal leads to glycolysis and the downregulation of mitochondrial membrane potential to promote γδ^IFN^ fate. Conversely, mitochondrial metabolism and high lipid content are indicative of γδ^17^ commitment. The effector lineage can also be defined by cell surface receptor expression, with γδ^IFN^ cells exhibiting a CD27^+^ CD45RB^hi^ CD44^int^ NK1.1^+^ profile, whereas γδ^17^ cells are CD27^neg^ CD45RB^neg^ CD44^hi^ CCR6^+^^23^^,^^28^^,^^29^ (Figure 2). Notably, a recent study using IL-17 and IFNγ reporter mice found that these surface markers fail to delineate pure γδ^17^ or γδ^IFN^ populations; therefore, transcriptomic analyses were used to identify new signature genes for these two effector populations.^28^ Surprisingly, the top 50 genes distinguishing γδ^17^ and γδ^IFN^ cells were not differentially expressed between Th17 and Th1 cells, which highlights the striking divergence of the transcriptome between the two γδ T cell subsets.

Within the gut mucosa, there is a distinct separation of Vγ expression and effector function between the two compartments. Vγ7 T cells comprise the majority of γδ IELs, with Vγ1 and Vγ4 TCRs expressed to a lesser extent, and all are γδ^IFN^ polarized. In contrast, IL-17-producing Vγ6 and Vγ4 T cells are predominant in the LP, along with the presence of a few IFNγ-producing Vγ1 and Vγ4 T cells.^30^ Given the segregation of Vγ chain usage and cytokine production profiles among γδ T cells in the epithelium and LP, it is presumed that each population remains restricted to its compartment. Although IELs likely migrate through the LP when initially trafficking to the epithelium, there is little evidence suggesting that γδ IELs freely exchange with γδ LPLs or vice versa. Finally, profiling of PPs revealed that most γδ T cells express the Vγ1Vδ6.3 TCR, with Vγ4 and Vγ6 T cells in the minority.^12^ Although 50% of the γδ T cells were found to express Vγ7 in this study, it is possible that these were contaminating IELs.

Despite their compartmentalization, the factors influencing γδ T cell homing to the gut are strikingly similar. Recruitment to the gut occurs through chemotaxis via CCR9, which is expressed by γδ T cells, binding to CCL25 produced by intestinal epithelial cells.^31^^,^^32^ The induction of CCR9 also promotes the expression of other gut homing markers, including β_7_ integrin, which forms heterodimers with α_4_ or α_E_ integrin. α_4_β_7_ integrin binds to MAdCAM-1 expressed by endothelial cells in the small intestinal and colonic LP.^33^ Once in the gut, both γδ LPLs and γδ IELs subsequently downregulate α_4_β_7_ integrin, and γδ IELs instead upregulate α_E_β_7_ (CD103), a ligand for epithelial E-cadherin.^34-36^ Despite an incomplete understanding of the factors that govern the homing and retention of γδ T cells within PP, differential expression of integrins appears to contribute to the distinct separation of γδ T cells within the mucosa.

γδ T cells seed the IEL compartment around the time of weaning, and as noted above, the majority express the Vγ7 TCR.^37^ These TCRs are highly polyclonal yet the ligand for murine γδ IELs has yet to be identified. Vγ7 IELs require the expression of epithelial butyrophilin-like (BTNL) molecules 1 and 6 for their selection and maintenance. BTNL1-deficient mice fail to establish a Vγ7 population, yet Vγ7 T cells are able to seed the epithelium but fail to expand following inducible deletion of BTNL1. Notably, Vγ7 IELs can develop in athymic mice and those lacking PPs, peripheral lymph nodes and MLN, suggesting that the local microenvironment is critical for shaping the γδ IEL compartment. Along these lines, epithelial BTNL expression is not dependent on dietary antigen or microbiota but is regulated by the epithelial transcription factors hepatocyte nuclear factor 4 (HNF4) *α* and γ.^37^^,^^38^ HNF4 family members play overlapping roles within the intestinal epithelium, yet HNF4α appears to contribute more to BTNL expression in the colon, whereas HNF4γ regulates its expression in the small intestine.^38^^,^^39^ Although Vγ1 and Vγ4 T cells are also found within the IEL compartment, the mechanism by which these subsets are specified has yet to be discerned.

In contrast to IELs, murine LP γδ^17^ cells exclusively develop in the embryonic thymus. The thymic development of γδ^17^ cells occurs within a window between day E15.5 and E18.5, and includes semi-invariant Vγ6Vδ1, Vγ4, and to a lesser extent, Vγ1 T cells.^18^ Despite a short developmental timeframe, γδ^17^ cells are long-lived and capable of self-renewal, as γδ^17^ cell number peaks in perinatal mice.^40^^,^^41^ There is also a small population of γδ LPLs that retain the ability to produce IFNγ.^4^ Together, these findings suggest that a substantial proportion of the γδ LPL compartment develops prenatally, which aligns with the innate functions of γδ^17^ cells.

Conventional T cell populations located either within the epithelium or LP are severely compromised in the absence of an intact microbiota.^2^ Since the development and homing of conventional T cells requires MHC-dependent activation, loss of commensal signals results in an underdeveloped immune response. Conventional TCRαβ IELs expand following exposure to commensal antigens,^42^ accumulating as mice age due to increased antigen exposure over time.^8^ In contrast, γδ IEL development occurs independent of the microbiota as gnotobiotic, or germ-free (GF), mice exhibit an intact γδ IEL compartment.^43^^,^^44^ Despite this, the seeding of γδ IELs is delayed in GF mice compared to conventional mice^45^ and antibiotic treatment of mice for six weeks immediately after birth leads to a reduction in γδ IEL number^46^ suggesting that commensal bacteria contribute to γδ IEL maintenance. Since many of the LP Vγ subsets develop embryonically and there are significantly fewer γδ LPLs than IELs under homeostatic conditions, the contribution of the microbiota to γδ LPL homing remains poorly understood. In the following sections, we will explore the contribution of the microbiota to the maintenance and function of γδ T cells within each compartment.

## Commensal factors support γδ IEL homeostasis and effector function

### Maintenance of the γδ IEL compartment

Whereas BTNL expression is essential for specifying Vγ7 IELs, IL-15 signaling is required to maintain all γδ IEL subsets. IL-15 is complexed with IL-15Rα and trafficked to the cell surface, where it is presented in *trans* to IL-2Rβ (CD122), which is expressed on γδ IELs.^47^ Mice deficient in IL-15, IL-15Rα or IL-2Rβ exhibit a reduced γδ IEL number due to decreased IEL proliferation and increased cell death.^48^^,^^49^ Many factors regulate IL-15 expression in the intestinal mucosa. For example, in addition to regulating BTNL expression, HNF4 family members also induce the expression of epithelial *Il15* and *Il15ra.*^39^ Toll-like receptor (TLR) and nucleotide-binding oligomerization domain-like receptor (NLR) signaling can also induce IL-15 production in epithelial cells or APCs, respectively. Although γδ IELs exhibit limited TLR expression,^50^ depletion of TLR2^51^ or its adaptor protein MyD88^49^^,^^52^ leads to a decrease in both small intestinal and colonic γδ IELs due to impaired epithelial IL-15 production.^31^ Moreover, the loss of APC-derived NOD2 similarly impairs the maintenance of small intestinal and colonic γδ IELs by reducing IL-15 production.^46^ Notably, supplementation with muramyl dipeptide (MDP), a NOD2 ligand, can rescue the γδ IEL population following antibiotic treatment.

In addition to innate immune activation by microbe-associated molecular patterns (MAMP), commensals such as *Clostridium*, *Bacteroides*, and *Lactobacillus* generate tryptophan-derived metabolites that serve as ligands for the aryl hydrocarbon receptor (AHR). AHR signaling is essential for maintaining small intestinal γδ IELs, as these cells are substantially reduced in mice with a germline or lymphoid-specific deletion of AHR, demonstrating a requirement for cell-intrinsic AHR signaling.^53^ Whereas the presence of commensal bacteria promotes epithelial AHR expression, it has yet to be determined whether IEL AHR is similarly regulated. However, the expression of AHR repressor (AHRR), a negative regulator of AHR, prevents lipid peroxidation and subsequent IEL ferroptosis.^54^ Taken together, these studies indicate that AHR/AHRR signaling must be tightly regulated to ensure IEL homeostasis.

### Purposeful restraint of γδ IEL effector function

**Figure 3.**
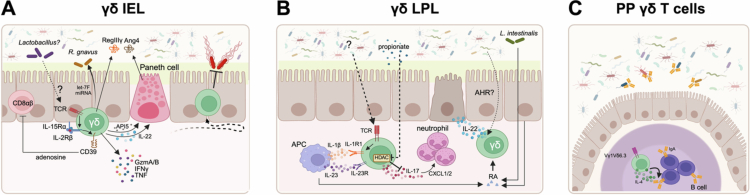
Compartmentalized roles of intestinal γδ T cells. (A) Regulatory γδ IELs express high levels of CD39, an ectonucleotidase that contributes to adenosine production. Microbiota such as *Lactobacillus* spp. induce TCR signaling to upregulate IL-15 signaling through IL-2Rβ, leading to enhanced CD39 expression. γδ IELs have the capacity to produce proinflammatory cytokines in addition to releasing granzymes. Further, γδ IELs influence the gut microbiota by producing let-7F miRNAs to enhance *R. gnavus* colonization, secrete antimicrobial peptides (AMP), and support Paneth cell function. To ensure optimal protection against microbial invasion, γδ IELs are highly motile and continuously survey the epithelium. (B) Antigen-presenting cell (APC)-derived IL-1β and IL-23 induce IL-17 production by γδ LPLs, which in turn can promote the recruitment of neutrophils into the LP. The microbiota is thought to induce TCR signaling to enhance IL-1 responsiveness through the upregulation of IL-1R1. Notably, the short chain fatty acid propionate can decrease IL-17 production by inhibiting HDAC activity. Retinoic acid (RA) is produced by APCs, epithelial cells, or commensal bacteria such as *L. intestinalis*, and contributes to γδ LPL IL-22 production, which facilitates tissue repair and contributes to the generation of AMPs. (C) Vγ1Vδ6.3 T cells are located within germinal centers of the Peyer's patch (PP) and produce IFNγ and IL-4, the latter of which promotes IgA class switching in B cells. Once translocated across the epithelium, commensal-specific IgA serves to limit microbial invasion and shape the composition of the microbiota. Created in BioRender.

It is challenging to specifically address the functional role of γδ IELs, as IEL-specific knockout mice have yet to be developed. Instead, many studies have used global *Tcrd* knockout mice to address the overall contribution of γδ T cells to intestinal physiology, but many past studies have failed to identify whether the observed phenotype is due to γδ IELs or LPLs. As noted above, γδ IEL activation can occur in an MHC-independent manner. γδ IELs express natural killer (NK) receptors, many of which are inhibitory, yet others, such as NKG2D, bind to epithelial stress ligands Rae-1 and H60 in mice or MICA and MICB in humans.^8^ The sensing of stressed enterocytes promotes IEL-mediated cytolysis of infected or damaged cells. At steady-state, γδ IELs express large amounts of the serine proteases, granzyme (Gzm) A and GzmB, which, when secreted in lytic vesicles with perforin, induce apoptosis (Figure 3A). While Gzms are typically associated with perforin-dependent cell death, CD103 ligation to epithelial E-cadherin was recently shown to promote the extracellular release of GzmA and B by γδ IELs to facilitate epithelial cell shedding into the lumen via a perforin-independent pathway.^55^ Moreover, IEL-derived GzmA promotes epithelial pyroptosis in response to *Salmonella* infection, whereas GzmB induces epithelial apoptosis.^56^ Although the increase in IEL-mediated cell death aids in clearing the infection, the increased availability of nutrients from apoptotic cells further promotes bacterial growth within the gut lumen.^56^^,^^57^

γδ IELs can secrete IFNγ and TNF, as well as chemokines involved in promoting T cell, NK cell and myeloid cell recruitment, including XCL1, CCL3, CCL4 and CCL5.^50^ Despite being associated with a pro-inflammatory or cytotoxic profile, γδ IELs are considered to be protective within the mucosa. γδ IELs prevent the translocation of commensal and pathogenic microbes through the production of antimicrobial peptides such as RegIIIγ, which is dependent on epithelial MyD88 signaling.^58^ Since γδ IEL PRR signaling is minimal, it is worth noting that most of what we know about commensal/γδ IEL crosstalk occurs with the intestinal epithelium serving as an intermediary. While we will expand on this point below, surprisingly little is understood regarding the mechanisms by which epithelial MyD88 signaling elicits γδ IEL effector function beyond IL-15 induction. γδ IELs can also produce various interferons through TCR-dependent and -independent mechanisms,^59^^,^^60^ highlighting the ability of these cells to bridge innate and adaptive immunity. Finally, γδ IELs secrete growth factors including keratinocyte growth factor (KGF), to promote epithelial proliferation at steady-state and in response to injury.^61-63^.

Unlike peripheral T cells or those in the LP, IELs are unique in that they exhibit an ‘activated, yet immunologically quiescent’ phenotype.^64^^,^^65^ At steady-state, IELs express many of the cell surface markers associated with T cell activation, including CD44 and CD69,^8^ which reflects the degree to which these sentinels are maintained in a poised state and are ready to respond to infection or insult. While the transcriptional profile of IELs supports a strong effector phenotype, proteomic studies indicate that γδ IELs exhibit reduced translational capacity.^65^ This is one of many ways in which IEL cytolytic effector function is restrained to avoid aberrant activation and potential tissue damage. In support of this, γδ IELs are significantly more refractory to TCR activation compared to conventional T cells; this can be partially attributed to the downregulation of several components of the TCR signalosome.^60^^,^^65^ Further, γδ IELs express a variety of inhibitory receptors^50^ such as LAG3, in addition to the CD8αα homodimer, which, when bound to the thymus leukemia (TL) antigen on epithelial cells, inhibits IEL activation and proliferation.^66^ In addition to the negative regulation of proximal TCR signaling events, IEL metabolic function is significantly constrained relative to other T cell populations.^67^ In a microenvironment in which epithelial cells are constantly bombarded with signals from luminal microbes, multiple mechanisms serve to dampen γδ IEL effector responses, ensuring that these sentinel lymphocytes can mount an appropriate response to stress and other insults while also limiting their potential for overactivation.

### γδ IELs serve as intestinal border patrol

Previous dogma suggested that γδ IELs were non-motile,^68^ presenting a model in which IELs migrated into the epithelium where they remained embedded between enterocytes. However, intravital imaging revealed that γδ IELs migrate dynamically along the basement membrane, moving in and out of the lateral intercellular space (LIS) between adjacent epithelial cells.^69^^,^^70^ Thus, γδ IELs can interact with multiple enterocytes within a short period to provide real-time surveillance of the epithelial barrier. This motility is regulated by direct interactions between the IEL and epithelium, including occludin, whose expression is required by both cell types for overall γδ IEL motility and CD103/E-cadherin ligation, which modulates the amount of time a γδ IEL is retained within the LIS.^69^ The loss of CD103 increases γδ IEL speed owing to the reduced dwell time in the epithelium.

Although γδ IELs are constantly patrolling, as a whole, γδ IELs preferentially localize in the mid-villus where they either migrate up toward the villous tip or down to the top of the crypt.^71^ However, in GF mice, γδ IELs are found lower on the crypt/villus axis with reduced overall motility. Similarly, γδ IEL migratory speed and migration into the LIS is decreased in conventional mice following treatment with broad-spectrum antibiotics.^71^^,^^72^ γδ IEL surveillance behavior was partially restored following segmented filamentous bacteria (SFB) monocolonization yet dual colonization with *Bacteroides vulgatus* and *E. faecalis* had no effect on IEL motility.^71^ The precise mechanisms by which the microbiota influences γδ IEL localization and migration has yet to be determined, but γδ IEL migratory dynamics were similarly compromised in both GF mice and those with an inducible depletion of epithelial MyD88. Thus, epithelial recognition of commensal bacteria appears to influence not only γδ IEL homeostasis but also their ability to survey the barrier.

While it is evident that γδ IELs respond to the recognition of commensals, γδ IEL migration into the epithelium is enhanced following the addition of the luminal pathogen *Salmonella* Typhimurium. γδ IELs migrate toward areas of bacteria-adjacent enterocytes or ‘hotspots’ of infection, which directly correlates with the ability of γδ IELs to limit microbial translocation across the epithelium.^70^^,^^71^ Inhibiting or enhancing γδ IEL surveillance via disruption of the molecular interactions described above resulted in an increase or decrease in translocation events, respectively.^70^ Again, epithelial MyD88 signaling contributed to this altered migratory behavior in response to *Salmonella* infection.^71^ PRR-induced IL-15 may also be involved in basal γδ IEL motility, as IL-15 signaling is required for PI3K-mediated polarity cues needed for γδ IEL surveillance behavior.^73^ We previously found that compartmentalized IL-15 overexpression within the gut mucosa directly impacted γδ T cell localization in the epithelium and LP. As expected, overexpression of epithelial IL-15 promoted γδ IEL proliferation and enhanced γδ IEL motility, whereas LP IL-15 overexpression led to an expansion of γδ LPLs. Blocking IL-2Rβ or drawing γδ T cells out of the epithelium and into the LP resulted in increased pathogen invasion. These findings indicate that the recruitment of γδ IELs to sites of bacterial attachment or invasion is likely necessary for the targeted secretion of antimicrobial factors by γδ IELs to limit microbial penetration and subsequent systemic dissemination.

### Effects of specific commensals on γδ IEL biology

Unlike conventional T cell populations,^74^^,^^75^ no single commensal species has been associated with a distinct γδ IEL phenotype. Recently, it was shown that a polyspecific TCRγδ recognizes indole-containing biomolecules,^76^ including indole-3-propionic acid (IPA), which are produced mainly by *Clostridia* species such as *Clostridium sporogenes.*^77^ Although this polyspecific TCR appears to only be expressed by a small proportion of γδ IELs in naïve mice, this finding raises the question of whether IPA-mediated activation of the TCR or AHR also contributes to γδ IEL homeostasis.

As noted earlier in the context of epithelial MyD88, indirect microbial sensing supports various IEL functions. Colonization of GF mice with *B. fragilis* or *B. thetaiotamicron* led to the recruitment of IL-6-producing colonic IELs in a MyD88-dependent manner to promote barrier integrity,^78^ yet how much of this response can be attributed to γδ IELs remains unclear. Our laboratory recently identified a unique microbiota that not only promotes the expansion of the γδ IEL compartment but also increases its surveillance behavior to limit systemic *Salmonella* infection.^72^ This hyperproliferative phenotype was only observed in the small intestine and was strongly associated with a guild of 7 amplicon sequence variants (ASV) in small intestinal luminal contents; the four most abundant from *Lactobacillus*, and one each from *Enterococcus*, *Faecalibaculum* and Muribaculaceae. We recently reported that this γδ IEL hyperproliferative-associated microbiota induces the expression of CD39, an ectonucleotidase that contributes to γδ IEL regulatory function,^39^ through TCR-mediated IL-15 signaling^79^ (Figure 3A). Notably, the highest levels of mucosal IL-15 and CD39 expression on γδ IELs were observed in the duodenum in both WT mice and those exhibiting the hyperproliferative phenotype,^79^ yet the composition of the luminal microbiota was not strikingly different between regions of the small intestine.^72^ Broad spectrum antibiotic treatment abrogated the increase in CD39 yet had no effect on γδ IEL number or mucosal IL-15 production, suggesting that continuous exposure to the microbiota may only contribute to certain aspects of the observed phenotype.^79^ Depletion of Gram-positive bacteria that produce indoles had no effect on the upregulation of CD39; thus, the specific bacteria or bacteria-derived molecules involved in regulating γδ IEL CD39 expression remain unknown. Given that epithelial cells often mediate crosstalk between the microbiota and γδ IELs, it would be of interest to determine whether microbial ligands directly activate the TCR or if this activation is indirect. Since antibiotics abrogated the increase in CD39 but not γδ IEL proliferation or migration, it is also possible that these commensals may induce long-term epigenetic changes to influence γδ IEL homeostasis.

### Role of IELs in intestinal secretory cell biology and microbiota regulation

A reciprocal relationship between the gut microbiota and the IEL compartment is essential for the activation of host immunity and barrier defense. For example, γδ IELs contribute both directly and indirectly to antimicrobial peptide (AMP) production (Figure 3A). At steady-state, the translocation of pathobionts across the barrier triggers epithelial MyD88 signaling, which is required for RegIIIγ production by small intestinal γδ IELs.^58^ In contrast, γδ IELs secrete RegIIIγ in a MyD88-independent manner in response to colonic injury in addition to lysozyme and complement proteins.^80^ γδ IELs also influence the biology of Paneth cells, the primary epithelial cell type responsible for AMP production. γδ IEL release of apoptosis inhibitor 5 (API5) promotes the viability of Paneth cells lacking the CD susceptibility gene *ATG16L1.*^81^ Moreover, IL-22 produced by γδ IELs is thought to promote Paneth cell expression of angiogenin-4 (Ang4).^82^ Mice deficient in γδ T cells exhibit fewer goblet cells, resulting in reduced mucin expression and glycosylation throughout the intestine.^83^ Consequently, this leads to a decrease in mucosal sialic acid, a nutrient source critical for mucosa-associated bacterial niche formation.^84^^,^^85^ γδ IELs also secrete microRNA let-7F to enhance the colonization of specific bacteria, such as the mucus-degrading *Ruminococcus gnavus.*^86^ These studies further emphasize the multifaceted role of γδ IELs in regulating host-microbe responses within the mucosa.

## γδ LPLs provide an early response to gut microbes

### Maintenance and activation of γδ LPLs

Unlike the defining contribution of BTNL proteins in shaping the γδ IEL compartment,^37^ there is no specific molecular interaction that specifies the maintenance of γδ T cell subsets (Vγ1,4,6) within the LP. Once Vγ6 and Vγ4 T cells exit the embryonic thymus, STAT5 is required for γδ^17^ survival and turnover following migration into peripheral tissues.^87^ Interestingly, the majority of Vγ6 LPLs in both the ileum and proximal colon co-express RORγt and Tbet upon entry into the gut. These RORγt^+^ Tbet^+^ γδ LPLs are polyfunctional, meaning that they can produce both Th1 and Th17 cytokines. However, within the first 2 d of life, STAT5A signaling functions to induce RORγt expression and subsequent γδ^17^ differentiation, and to suppress Tbet, a transcription factor associated with Th1 signaling. STAT5 is activated downstream of cytokine receptor signaling, including IL-7, for which γδ LPLs express high levels of CD127 (IL-7R)^88^ (Figure 2B). In addition to STAT5, the cellular inhibitor of apoptosis protein (cIAP) 1/2 is required to sustain γδ^17^ cells later in the neonatal period through early life.^89^ Loss of cIAP1/2 leads to a reduction in the expression of the lineage transcription factors cMAF and RORγt and impairs γδ^17^ proliferation in response to cytokine. Antibiotic treatment partially rescues the loss of γδ^17^ LPLs in the absence of cIAP1/2 expression, suggesting that commensals may stress the already fragile cIAP1/2-deficient γδ^17^ cells.

In the distal colon, there is a small population of CCR6^+^ SCART2^+^ Vγ4 T cells that produce IL-17.^41^ These γδ^17^ cells are largely found within colonic ILFs with a sparse distribution in the LP and a negligible presence in the SI. Consistent with other γδ^17^ populations, these SCART2^+^ Vγ4 T cells are embryonically derived with a long lifespan and a slow turnover rate. While the functional contribution of these SCART2^+^ γδ^17^ cells remains unclear, it is possible that these cells may function to locally secrete IL-17 within the ILF or eventually migrate to populate the LP.

Whereas CD4 Th17 cells require TCR stimulation plus IL-23 and IL-1 for IL-17 induction, γδ T cells can produce IL-17 in response to these cytokines without TCR engagement.^90^ However, mice lacking IL-1R1 exhibit reduced γδ^17^ cells in the periphery, indicating that cytokine exposure is essential for an effector response. To this end, small intestinal γδ^17^ LPLs expressing IL-1R1 may comprise a resting population of innate effector cells that can be quickly activated by IL-23 and IL-1 in response to MAMP-mediated activation of APCs.^91^ Thus, it is not surprising that GF mice exhibit fewer activated IL-1R1^+^ γδ^17^ LPLs compared to SPF mice, emphasizing that colonization with commensals is required for the expansion of IL-1R1^+^ γδ LPLs. IL-1R1^+^ γδ LPL proliferation and their capacity to produce IL-17 are dependent on TCR signaling through the guanine nucleotide exchange factor Vav1, indicating that the microbiota stimulates the TCR to prime γδ^17^ activation (Figure 3B).

In addition to IL-1R1, a recent study showed that colonic CD44^hi^ γδ^17^ cells express high levels of PD-1 that was not directly associated with TCR signaling or a specific Vγ subset.^92^ Treatment with broad-spectrum antibiotics reduced PD-1 expression. Diminished PD-1 levels correlate with decreased IL-17 production in endogenous γδ^17^ LPLs, yet these cells retain the ability to produce cytokines in response to *ex vivo* stimulation. Conversely, short-term PD-1 blockade increased the number of colonic γδ^17^ effector cells. Together, these findings indicate that commensals upregulate PD-1 expression on γδ^17^ cells to modulate the amount of IL-17 produced *in vivo.* The commensal factors involved in PD-1 upregulation remain unclear^92^ as previous attempts to define the specific microbes needed to maintain γδ^17^ LPL populations did not yield candidate bacteria.^91^ Notably, the frequency of IL-1R1^+^ γδ^17^ cells was reduced following treatment with neomycin or vancomycin, but no change was observed in those receiving metronidazole; this suggests that facultative Gram-positive and/or Gram-negative microbes are involved. Collectively, these findings demonstrate that initial exposure to a commensal antigen may activate the TCR to drive the expansion of IL-1R1^+^ γδ^17^ LPLs and that the subsequent production of IL-23 and IL-1 by MAMP-activated APCs is sufficient to elicit an innate γδ^17^ response that is regulated via PD-1 expression.^90-92^

Although the microbiota is a key contributor to IL-17 and IL-22 production by small intestinal γδ LPLs, the opposite is observed in the cecum and colon.^93^ These findings suggest that differences in microbiota composition may influence how γδ LPL effector function is shaped. For example, γδ^17^ LPLs are increased and produce more IL-17 in the ceca of GF mice, a phenotype also observed in broad-spectrum antibiotic-treated SPF mice. Further investigation revealed that vancomycin treatment had opposing effects on regional γδ^17^ LPL populations; Gram-positive bacteria were required for γδ^17^ LPLs in the small intestine but repressed these cells in more distal regions of the gut.^91^^,^^93^^,^^94^ Short chain fatty acids (SCFAs), including acetate, butyrate, and propionate, are synthesized primarily by *Firmicutes* and *Bacteroidetes* through the fermentation of dietary fiber.^95^ In particular, propionate was found to negatively influence cecal γδ^17^ number along with IL-17 production via inhibition of histone deacetylase activity.^93^ These findings indicate that the local microenvironment and presence of individual commensal species likely contribute to the specification and maintenance of γδ^17^ LPL populations (Figure 3B).

As mentioned earlier, *C. sporogenes* can generate IPA,^77^ which is recognized by a small subset of γδ LPLs expressing a polyspecific TCRγδ in naïve mice.^76^ It remains unclear whether TCR recognition of IPA contributes to intestinal γδ T cell homeostasis in the gut; however, IPA contributes to the maintenance of innate-like Vγ6Vδ1 T cells in the lung and regulates γδ^17^ T cell expansion during infection. Thus, it is possible that Gram-positive commensals may support γδ^17^ LPL expansion and function through multiple mechanisms.

### γδ LPL effector function and role in infection

γδ LPLs are considered to be protective under homeostatic conditions, as their ability to produce IL-17 induces an epithelial antimicrobial response and promotes neutrophil recruitment via the induction of CXCL1 and CXCL2.^41^^,^^96^^,^^97^ In early life, IL-17-producing Vγ6 LPLs mount an innate response to *Clostridium difficile* infection, with expansion of γδ^17^ cells in the MLN observed within 2 d post-infection and peak γδ^17^ LPL number observed in the cecum and colon 4 d later.^96^ This early response is critical for host defense, as γδ T-cell-deficient mice were more susceptible to *C. difficile* infection. Moreover, a similar positive correlation between γδ T cells and *IL17A* transcripts was reported in children with *C. difficile* infection relative to controls.

*Listeria monocytogenes* (*Lm*) infection induces a population of memory γδ T cells that produces both IFNγ and IL-17.^98^ These CD27^neg^ CD44^hi^ Vγ6Vδ1 T cells express similar levels of RORγt compared to other γδ^17^ cells but also exhibit high Tbet expression. *Lm*-induced memory γδ T cells upregulate α_4_β_7_ integrin to support homing to the LP, where these cells eventually contract after the primary infection and quickly become reactivated following secondary challenge. Further, these memory γδ LPLs exhibited a more robust response compared to memory CD8 T cells. Despite expressing both IFNγ and IL-17 after oral *Lm* infection, most memory γδ T cells expressed only IL-17, with a smaller proportion retaining polyfunctionality. This γδ^17^ memory response required both MHCII^+^ APCs and TCR signaling, with antibody-mediated TCR internalization leading to reduced IL-17 production and loss of protection against infection. However, these memory Vγ6Vδ1 T cells were generated in GF mice, demonstrating that their development did not require an intact microbiota.^99^ Surprisingly, contact with other enteric bacteria, including *S. Typhimurium* and *C. Rodentium* activated a delayed TCR-mediated polyfunctional response in memory Vγ6Vδ1 T cells, indicating a degree of promiscuity in their ability to mount a recall response to enteric pathogens.

γδ LPLs express IL-17 and IL-22 under homeostatic conditions and thus exhibit functional overlap with type 3 CD4 T cells. One main difference is that Th17 cells require antigen exposure to differentiate, a process that occurs over several days, whereas γδ^17^ cells are more innate-like and respond quickly to stimulation.^90^ Not only is this an advantage in terms of a temporal response, but γδ^17^ cells dominate early life responses since they develop before Th17 cells.^100^ Lastly, γδ^17^ cells are also more effective producers of IL-17, secreting more cytokine on a per cell basis compared to Th17 cells.^90^ While the contribution of IL-17 produced by γδ LPLs is apparent, the relative contribution of γδ LPL-derived IL-22 in host defense or tissue repair is less clear since type 3 ILCs are the main IL-22 producers in the LP.^101^^,^^102^ IL-22 promotes both epithelial regeneration and the production of AMPs to prevent microbial translocation across the barrier.^103^^,^^104^ AHR signaling induces IL-22 expression, yet whether this occurs in γδ LPLs and if AHR-mediated IL-22 production is IL-23-dependent in γδ T cells has yet to be determined.^102^^,^^105^^,^^106^

## γδ T cells in Peyer's patches

Mice with a germline deletion of γδ T cells exhibit a marked reduction in the frequency of IgA^+^ B cells and reduced serum and fecal IgA.^107^ While the contribution of γδ T cells in helping B cell antibody production has been well-documented, less is known regarding the role of γδ T cells in PPs.^108^^,^^109^ Vγ1Vδ6.3 T cells are located within the germinal centers (GC) and secrete IL-4 to induce GC B cell class switching to promote IgA production^12^ (Figure 3C). Within the lumen, secreted IgA binds to commensals and helps shape the composition of the microbiota.^110^^,^^111^ Consistent with prior reports, inducible depletion of γδ T cells has no effect on epithelial barrier function or microbiota composition; however, GC organization was disrupted and there was a reduction in GC B cell proliferation in the absence of γδ T cells.^12^ Notably, TCR signaling was not required for IL-4 production or the frequency of IgA^+^ B cells, suggesting that the function of PP γδ T cells may be TCR-independent and facilitated by constitutive exposure to commensal bacteria. Relatively little is known about when these γδ T cells populate the GCs or the commensal signals that contribute to their activation, making this an exciting area for future investigation.

## Compartmentalization of γδ T cells and IBD pathogenesis

Inflammatory bowel disease (IBD) encompasses both Crohn's disease (CD) and ulcerative colitis (UC), which are chronic inflammatory diseases of the digestive tract.^112^ Whereas UC affects only the colon, inflammation associated with CD can occur anywhere along the gastrointestinal tract. Although the exact etiology is unknown, loss of epithelial barrier function, aberrant mucosal immune activation, and microbial dysbiosis contribute to disease development. An excessive response to commensal bacteria coupled with a disproportionate increase of effector immune cells in relation to regulatory cells promotes the development of a pro-inflammatory microenvironment. The inflammation associated with IBD results in a shift in the composition of the microbiota, characterized by a loss of *Firmicutes* and SCFA producers and an increase in *Proteobacteria.*^113^^,^^114^ A recent study identified a preclinical microbiome signature within a cohort of healthy first-degree relatives of IBD patients, indicating that dysbiosis may play a causal role in the pathogenesis of CD.^115^ The use of experimental colitis models showed that γδ T cells are largely protective against disease development, as γδ T-cell-deficient mice exhibit increased susceptibility to both 2,4,6-trinitrobenzene sulfonic acid (TNBS) and dextran sodium sulfate (DSS)^116-118^ (Table 1). Adoptive transfer of peripheral γδ T cells into TNBS-treated mice mitigated the severity of colitis through the production of IL-10 and TGFβ.^119^ However, a critical limitation in the interpretation of these studies is that the use of mice with a global deletion of γδ T cells fails to clearly identify which γδ T cell compartment contributes to protection against inflammation.

**Table 1.** Role of γδ T cells in different animal models of IBD.

Disease model	Experiment	Compartment studied	Effect on disease outcome	Mechanism of action	Reference
TNBS-induced colitis	Adoptive transfer of peripheral γδ T cells	N/A	Protective	γδ T cells promote IL-10 and TGFβ production	[119]
	Tcrd KO mice	N/A	Protective	Not described	[116,117]
DSS-induced injury	Tcrd KO mice	N/A	Protective	Not described	[118]
	Neutralization of IL-17	LP	Protective	IL-17 promotes barrier integrity and inhibits immune recruitment to gut	[120,121]
	Adoptive transfer of γδ^17^ cells	LP	Protective	IL-17 promotes recruitment of immunosuppressive CD11b^+^ myeloid cells	[122]
	IL-22-deficient mice; neutralizing IL-22	LP	Protective	IL-22-induced production of antimicrobial peptides	[102,123]
T cell transfer colitis	Adoptive transfer of CD103^+^ α_4_β_7_^hi^ γδ T cells	LP	Inflammatory	CD103^+^ α_4_β_7_^hi^ γδ T cells promote Th1/Th17 recruitment	[124]
	Adoptive transfer of peripheral γδ^17^ cells and CD4 T cells	LP	Inflammatory	γδ^17^ cells promote Th17 differentiation	[125]
TNF^ΔARE^ spontaneous ileitis	Longitudinal analysis of IEL compartment	IEL	Protective	Reduced γδ IEL number, CD39-mediated regulatory function, and surveillance prior to ileitis onset	[39]
	Tcrd KO mice	N/A	No change	N/A	[126]
	Anti-TCRγδ	N/A	Protective	Not described	[127]
SAMP/YitFC spontaneous ileitis	Longitudinal analysis of IEL compartment	IEL	Protective	Loss of γδ IELs prior to ileitis onset; no change in γδ LPL number	[39,128]
TCRα KO spontaneous colitis	Anti-TCRγδ	LP	Inflammatory	Pro-inflammatory IL-4-producing Vγ1 T cells expand, blocking TCRγδ reduces severity	[129]
	Cross to KN6 Tg	LP	Protective	Unknown	

Our understanding of intestinal γδ T cell development and effector function is largely derived from mouse models, as it is challenging to obtain sufficient cell numbers from human tissue biopsies for functional studies. In this section, we will discuss both the role of γδ IELs and LPLs in IBD and how these populations and/or their function is altered during disease. Since there are relatively few studies of intestinal γδ T cells in patients with IBD, we will focus on relevant IBD translational studies supported by data from experimental models to provide additional mechanistic context. We will also consider the reciprocal relationship between the microbiota and γδ T cell biology in the context of gut inflammation. Finally, we will expand on how the field may address the inherent challenges involved in validating and translating *in vivo* findings of γδ T cell biology to human disease.

### Role of γδ IELs in intestinal inflammation

Evaluation of the IEL compartment in patients with ileal CD revealed a loss of γδ IELs in both non-inflamed and inflamed tissue, which was similarly observed in patients with Crohn's colitis and UC.^130^^,^^131^ This depletion of Vγ4Vδ1 IELs, the human counterpart of murine Vγ7, is linked to diminished epithelial BTNL expression.^131^ Vγ4 IELs are re-established in healed ulcerations, and the sustained presence of γδ IELs correlated with disease remission,^131^ indicating that γδ IELs protect against intestinal inflammation. These studies using biopsies obtained from patients during active inflammation provide valuable information regarding mucosal immune populations; however, data collected from a biopsy only reflect a single snapshot in time. As it is challenging to obtain patient samples prior to disease initiation, animal models provide an opportunity to determine whether loss of γδ IELs is a cause or consequence of inflammation. Our group found that γδ IEL number is reduced three weeks prior to the onset of spontaneous CD-like ileitis in TNF^ΔARE/+^ mice.^39^^,^^132^ A similar reduction was observed early in the development of inflammation in SAMP/YitFc mice, another model of chronic ileitis,^128^^,^^133^ further indicating that the loss of protective γδ IELs may be an initiating factor in disease development. Previous studies regarding the role of γδ IELs in chronic ileal inflammation have yielded conflicting results.^17^^,^^18^ TNF^ΔARE/+^ mice with a germline deletion of γδ T cells were phenotypically similar to controls^126^; however, TCRαβ IELs can functionally compensate for the absence of γδ IELs.^134^ Anti-TCRγδ treatment of TNF^ΔARE/+^ mice led to more severe ileal pathology,^127^ but later studies found that this antibody only induces TCR internalization and fails to deplete γδ T cells.^135^ Thus, the various innate-like functions attributed to γδ IELs would not be impacted.^58^^,^^70^^,^^80-82^ To resolve this question, we generated TcrdGDL; TNF^ΔARE/+^ mice allowing for inducible depletion of γδ T cells;^39^^,^^136^ however, crossing the two strains resulted in the establishment of a microbiota that enhanced the severity of ileitis. Despite this confounding result, we observed that earlier loss of γδ IELs correlated with an earlier onset of disease.

**Figure 4.**
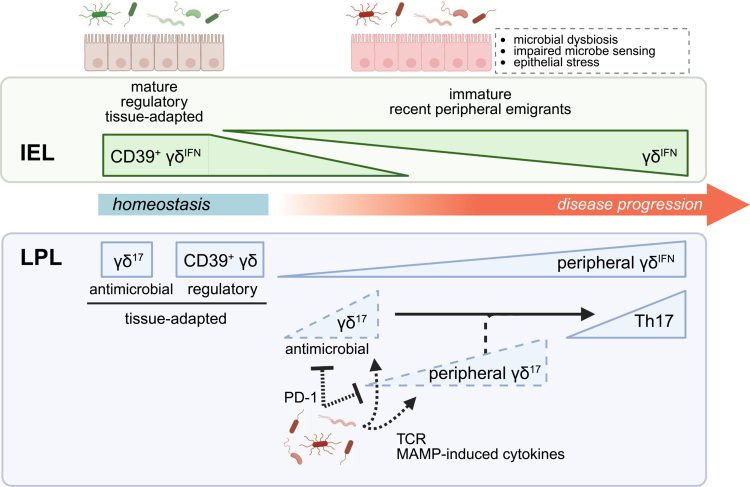
Proposed relationship between tissue-resident and peripheral γδ T cells in intestinal inflammation. Based on data obtained from patient studies and murine models of IBD, we propose that γδ T cell compartments undergo substantial changes during the onset and progression of disease. The mature, regulatory, IFNγ-polarized γδ IELs present under homeostatic conditions are substantially depleted prior to the onset of ileal inflammation, which coincides with an influx of peripheral γδ T cells that fail to mature within the epithelium. We posit that under inflammatory conditions, the epithelium may be unable to properly educate these immature γδ IELs, perhaps due to altered innate immune signaling or increased epithelial stress. The lamina propria is largely comprised of innate-like γδ^17^ T cells that expand following epithelial injury as part of an antimicrobial response. A population of regulatory CD39^+^ γδ LPLs is reduced in patients with IBD, whereas an increase in peripheral γδ^IFN^ T cells is observed. γδ^17^ LPLs are thought to promote Th17 expansion later in disease development; however, whether this is due to the expansion and activation of tissue-resident γδ^17^ LPLs or recruitment of IL-17-producing peripheral γδ T cells is not known. It is possible that the microbiota inhibits IL-17 production by γδ^17^ LPLs via upregulation of PD-1 or, alternatively, promotes their activation via TCR or cytokine signaling. Although these regulatory mechanisms may function as a rheostat for IL-17 production in the lamina propria at steady-state, dysregulation of this process either early or late in disease may further exacerbate inflammation. Created in BioRender.

In investigating the mechanisms underpinning γδ IEL loss, we observed that reduced expression of the epithelial HNF4γ/BTNL signaling axis led to depletion of Vγ7 IELs.^39^ Since HNF family paralogs regulate IL-15 expression^39^ which is essential for γδ IEL survival and motility, among many other cellular functions,^48^^,^^49^^,^^73^^,^^79^^,^^137^ it is not surprising that TNF^ΔARE/+^ mice exhibit increased γδ IEL apoptosis at early timepoints prior to ileitis onset.^39^ The motility of the remaining γδ IELs in TNF^ΔARE/+^ mice was significantly compromised, indicating that barrier surveillance was diminished before disease initiation. Notably, the loss of these mature γδ IELs corresponded with an influx of peripheral Vγ1 and Vγ4 T cells into the epithelium that failed to mature into bonafide IELs (Figure 4). Systemic depletion of the Vγ1 and Vγ4 T cells had no effect on either disease initiation or progression, suggesting that these immature recent emigrants fail to functionally replace the early loss of Vγ7 IELs. This migration of peripheral γδ T cells into the IEL compartment was similarly observed in both patients with UC and Crohn's colitis.^131^ Thus, in addition to a failure to support the maintenance of mature, tissue-resident γδ IELs, it appears that the local mucosal microenvironment is also unable to sufficiently educate peripheral γδ T cells during preclinical stages of disease.

Many microbial sensing pathways contributing to IEL homeostasis are impaired in IBD. Individuals expressing the CARD9 IBD risk allele exhibit defective AHR signaling,^15^ as do CARD9-deficient mice, leading to reduced tryptophan metabolism.^138^ Similarly, intestinal AHR expression is decreased in CD patients,^139^^,^^140^ and loss of AHR signaling decreases γδ IEL number and increases susceptibility to colonic injury.^53^^,^^54^ As described earlier, regulatory γδ T cells express CD39, an ectonucleotidase that contributes to the hydrolysis of extracellular ATP into adenosine,^141^ which inhibits T cell proliferation and proinflammatory effector function. CD39^+^ γδ IELs are decreased in pediatric IBD patients,^142^ and we recently noted a reduction in the frequency of CD39^+^ γδ IELs prior to ileitis onset in mice.^39^ While AHR activation promotes CD39 expression in CD4 IELs,^143^ whether a similar mechanism modulates γδ IEL regulatory function has yet to be described. One clinical study showed that pharmacological induction of CD39 alleviates pediatric colitis,^142^ identifying possible mechanisms by which commensals promote CD39 upregulation may prove to be a useful strategy to limit intestinal inflammation.

The innate immune sensor NOD2 was the first genetic risk factor identified for ileal CD,^144^^,^^145^ and its deletion correlates with a reduced γδ IEL number and increased susceptibility to inflammation.^46^^,^^146^ Moreover, NOD2-deficient mice were found to have an increase in IFNγ^+^ γδ IELs, which coincided with changes in goblet cell function, an expansion of the mucosa-associated commensal *B. vulgatus*, and enhanced susceptibility to inflammation.^146^ Importantly, NOD2 affects other natural IEL populations; therefore, the observed effect cannot be attributed solely to γδ IELs. Despite the strong connections between microbial sensing and γδ IEL biology, these studies have largely focused on enteric infections, leaving an opportunity for more studies relating to IBD.

Collectively, these studies suggest that multiple layers of γδ IEL dysfunction precede disease initiation, which provides valuable translational insight. First, identifying the underlying mechanisms leading to the downregulation of the HNF4/BTNL axis may clarify a disease-initiating trigger. These defects may be further compounded by the loss of innate signaling in individuals harboring NOD2 polymorphisms or other loss-of-function microbial sensing pathways. The knowledge that a reduction in γδ IEL number and/or regulatory function precedes intestinal inflammation could serve as a means to predict disease relapse or loss of drug responsiveness. Although it may be challenging to use tissue-based immune profiling as a diagnostic or predictive tool, increased homing of peripheral γδ T cells to the gut may serve as a surrogate measurement for a compromised γδ IEL compartment. Since the restoration of γδ IELs leads to sustained remission,^131^ it would be ideal to define novel approaches to either maintain these regulatory γδ IEL populations and/or promote peripheral γδ T cell differentiation into a more mature IEL phenotype. Pharmacological or microbiota-based approaches to upregulate CD39 expression on γδ IELs^79^^,^^142^ remain an open area for future investigation.

### Role of γδ LPLs in intestinal inflammation

Patients with active CD exhibit increased IL-17 within the LP,^147^ yet deletion or neutralization of IL-17 exacerbates disease severity in both animal models and patients^120^^,^^121^^,^^148-150^ pointing to a protective role for IL-17 in mucosal homeostasis. γδ T cells are the primary source of IL-17 in the LP within 3 d of DSS treatment,^121^ indicating the critical contribution of γδ^17^ LPLs to the early response to injury. To this end, IL-17 was found to promote barrier integrity by antagonizing tight junction protein internalization in response to pro-inflammatory cytokine. This early γδ^17^ response to acute colonic injury did not require IL-23; however, the contribution of IL-1 signaling was not investigated. Further longitudinal analysis revealed that the frequency of γδ^17^ LPLs gradually climbs and peaks after 10 d of DSS exposure, whereas a transient increase in the frequency of CD4 Th17 cells is observed at day 10.^122^ PD-1 expression was shown to restrain IL-17 production by colonic γδ^17^ cells, and surprisingly, PD-1 was further upregulated on these cells following 7 d DSS treatment.^92^ Similar findings were reported in Muc2-deficient mice,^92^ which develop spontaneous chronic colitis,^151^^,^^152^ indicating that microbial regulation of PD-1 also serves to regulate γδ^17^ activation during intestinal inflammation. Commensal bacteria promote a protective innate γδ^17^ response, as concomitant antibiotic and DSS exposure reduced both colonic γδ^17^ LPLs and neutrophil infiltration compared to mice receiving DSS alone.^153^ Despite the fact that propionate inhibits IL-17 production by cecal γδ LPLs,^93^ oral administration of this SCFA ameliorates DSS-induced injury.^154^ Thus, further studies are needed to determine how propionate affects colonic γδ^17^ cells following injury or if the beneficial effects observed are independent of γδ LPLs as propionate was also shown to improve barrier integrity and dampen pro-inflammatory cytokine production.

Several studies addressing the specific contribution of γδ LPL subpopulations to the pathogenesis of intestinal inflammation have yielded mixed results. Whereas transfer of peripheral CD27^+^ γδ^IFN^ T cells into γδ T-cell-deficient mice had no effect on DSS-induced injury, reconstitution of the γδ^17^ population was protective.^122^ In contrast, co-transfer of CCR6^+^ γδ^17^ cells and CD4 T cells resulted in more severe T cell transfer colitis compared to those mice receiving CCR6^neg^ γδ^IFN^ cells, suggesting that γδ^17^ cells support the differentiation of colitogenic Th17 cells during chronic inflammation.^125^ Taken together, the relative contribution of γδ^17^ LPLs may be dependent on whether an epithelial insult initiates inflammation or the composition of the adaptive immune compartment as suggested by the reported pathogenic contributions of γδ^17^ cells in T cell transfer experiments.

Mice deficient in TCRα develop spontaneous colitis driven by a Th2 inflammatory response.^155^^,^^156^ In this model, dysregulated IL-4-producing Vγ1 T cells expand within the colonic LP to induce a pro-inflammatory response in the absence of αβ T cells.^129^^,^^156^ Blocking TCRγδ signaling partially reduced the severity of colitis, indicating that this response is TCR-dependent. Notably, crossing TCRα KO mice to those expressing a transgenic Vγ4 TCR reduced the severity of disease, yet whether this is a result of fewer IL-4^+^ Vγ1 or more IL-17^+^ Vγ4 LPLs was not addressed.

In addition to IL-17, γδ LPL-derived IL-22 may also reduce the severity of gut inflammation.^102^ IL-22 is increased in the mucosa of patients with IBD^157^, and mice lacking IL-22 are more susceptible to colitis.^123^ IL-22 expression is enhanced by retinoic acid (RA), a vitamin A metabolite primarily produced by intestinal DCs. Moreover, the microbiota contributes to RA production through microbial or epithelial RA synthesis.^158^^,^^159^ RA administration ameliorated disease in DSS-induced injury and recovery and reduced inflammation in response to *C. rodentium* infection.^102^ Although ILC3s remain the dominant IL-22-producers in the gut at steady-state and in response to DSS,^153^ the identification of specific commensals and/or microbial factors that can enhance γδ LPL secretion of IL-17 and IL-22 may allow for an enhanced antimicrobial response early in disease development to limit the severity of intestinal inflammation.

One study identified a novel subset of CD103^+^ α_4_β_7_^hi^ γδ T cells that expanded upon CD4 T cell transfer into TCRβ-deficient mice and also developed following co-transfer with naïve CD4 T cells into RAG-deficient hosts.^124^ Further investigation revealed that these CD103^+^ α_4_β_7_^hi^ γδ T cells were IFNγ-producing and that the number of these cells in circulation correlated with the severity of T cell transfer colitis, yet CD103^+^ α_4_β_7_^hi^ γδ T cells were also observed within the colonic LP and MLN of SAMP/YitFc mice. These findings suggest that the expansion of a γδ^IFN^ LPL population may contribute to disease pathogenesis, further emphasizing the importance of a compartmentalized γδ^IFN^/γδ^17^ response within the gut mucosa.

The most well-characterized γδ T cells in humans are circulating Vγ9Vδ2 T cells that are activated by endogenous and microbial phosphoantigen via the TCR.^160^ Approximately 1%–5% of intestinal γδ T cells express Vγ9Vδ2; these cells are recruited from the periphery but proliferate locally and upregulate CD103 in response to microbial antigen.^161^ Upon exposure to the microbial phosphoantigen HMB-PP produced by many commensals,^162^^,^^163^ Vγ9Vδ2 LPLs upregulate MHCII and may function to stimulate IL-22 production in CD4 LPLs.^164^ In patients with IBD, these Vγ9Vδ2 T cells are recruited from the periphery and accumulate in the LP where they exhibit both pro-inflammatory and protective properties.^161^^,^^164^^,^^165^ The majority of Vγ9Vδ2 T cells secrete IFNγ; however, there is a population of CD28^+^ CD27^+^ CCR6^+^ cells that express IL-17 in inflamed tissue, despite the rarity of γδ^17^ cells in healthy individuals.^166^^,^^167^ Consistent with this, increased gut homing of CD27^+^ Vδ2 T cells that produce TNF and IL-17 has been observed in patients with CD.^168^

Colonic CD39^+^ Vδ2^neg^ LPLs display a tissue resident memory phenotype and secrete IL-10 with reduced IL-17 and IFNγ expression.^169^ Notably similar to their IEL counterparts,^39^ the frequency of CD39^+^ γδ LPLs is also markedly reduced in IBD.^169^ The regulation of γδ LPL effector function within the mucosa in IBD is significantly understudied. It is possible that tissue-resident γδ LPL subsets exhibit varying degrees of sensitivity to microbial antigens or recognize entirely different microbial signals compared to peripheral Vγ9Vδ2 T cells that have migrated into the LP. Whether commensal-derived factors influence the effector function of these different γδ LPL subsets warrants additional investigation.

While many questions remain regarding the contribution of human γδ^17^ LPLs to IBD, current literature indicates that murine γδ^17^ LPLs may have a biphasic role in the context of intestinal injury and inflammation. Consistent with their role in innate immunity, γδ^17^ LPLs likely protect against disease initiation and progression, whereas these cells may support colitogenic Th17 differentiation later in disease pathogenesis. Commensal bacteria clearly contribute to the γδ^17^ LPL activation; however, mechanistic studies in this area have gained traction only in recent years. Since global inhibition of IL-17 signaling results in worse patient outcomes,^148^^,^^149^ a clearer understanding of the temporal contribution of γδ^17^ LPLs to disease pathophysiology is required. Thus far, it appears that the expansion of γδ^IFN^ LPLs elicits a pro-inflammatory response, yet there are a dearth of studies in this area as well.

### Future avenues for translational studies of γδ T cells in IBD

As noted above, obtaining sufficient numbers of human mucosal γδ T cells for experimental assays is often challenging. A handful of single-cell transcriptomics datasets that include intestinal mucosal γδ T cells are available and provide a good starting point to investigate γδ T cell profiles in IBD.^130^^,^^170-174^ Moreover, the growing interest in spatial transcriptomics will allow for a more precise evaluation of γδ T cells within their native compartment.^175^ However, there is a pressing need to develop more tractable means to investigate how host-microbiota interactions shape γδ T cell biology. Our group has shown that *ex vivo* culture of murine γδ IELs results in the loss of the IEL phenotype and the development of a functional profile more consistent with that of conventional CD8 T cells.^60^ While this phenomenon has not been investigated in human γδ IELs, it stands to reason that co-culture with epithelial cells may allow for their expansion while maintaining tissue-adapted characteristics. Recent studies have reported the successful co-culture of human T cells (IELs and LPLs) with human organoids.^176^^,^^177^ Further, the ability to culture human intestinal organoids in monolayers simplifies studies to apply luminal microbes or microbial products to the apical surface of the epithelium relative to a 3D co-culture. Alternatively, expanded use of gut-on-a-chip models may be particularly useful to address questions relating to γδ LPL biology, as this experimental system allows microbes to be added to the apical chamber and immune cells to be perfused into a lamina propria compartment.^178^ It is possible that a gut-on-a-chip methodology could also be used to investigate IEL biology; however, to our knowledge, this has yet to be demonstrated.

## Perspectives and future directions

In summary, intestinal γδ T cells across all three compartments contribute to the maintenance of mucosal homeostasis and/or provide a rapid response to enteric pathogens. Further, γδ T cell activation status and effector function are finely tuned based on the local microenvironment. Although substantial crosstalk exists between the microbiota and these γδ T cell populations, additional studies are needed to discern the underlying mechanisms by which commensal bacteria shape and support γδ T cell function. In regard to γδ IELs, downstream signaling following microbial sensing is largely mediated through the epithelium. Further investigation of epithelial zonation^179^ may highlight regional transcriptional profiles that correlate with microbial composition or density, which in turn could translate into regional differences in γδ IEL/epithelial crosstalk. Likewise, spatial biology has the potential to define the cellular neighborhoods occupied by gut γδ T cells and to what extent depletion of the microbiota, inflammation-induced dysbiosis, or diet alter γδ T cell functionality within each compartment.

Our data indicate that early γδ IEL dysfunction may trigger or exacerbate IBD pathophysiology; thus, it would not be surprising if similar processes occurred in other mucosal compartments. For example, regulatory γδ T cells are depleted from both the epithelium and LP in patients with IBD.^130^^,^^142^^,^^169^ Data obtained from both human samples and murine models reveal that mature γδ IELs are replaced by peripheral γδ T cells during inflammation. Based on their broad functionality, the migration of peripheral γδ T cells into a pro-inflammatory microenvironment may induce a phenotype distinct from that of their original tissue-resident counterparts (Figure 4). While this is documented within the IEL compartment,^39^ the impact of peripheral γδ T cell infiltration into the LP has yet to be determined. We hypothesize that during the initiation and progression of intestinal inflammation: (1) tissue-resident γδ^17^ LPLs may be dysregulated, thus inhibiting a key early antimicrobial response; (2) an influx of γδ^IFN^ cells promotes a more pro-inflammatory microenvironment; (3) an altered microbiota dampens γδ^17^ LPL effector function; and/or (4) the recruitment of peripheral or prolonged activation of tissue-resident γδ^17^ LPLs drives an inflammatory Th17 response. Moreover, additional studies are needed to understand whether commensals contribute to γδ^17^ LPL activation via TCR- or MAMP-mediated signaling during disease development or, alternatively, suppress their effector function through PD-1 upregulation. The paucity of data on PP γδ T cells also leads to questions as to whether these cells remain within PPs during disease or egress into the LP, thus compromising IgA production.

In this review, we have discussed how the microbiota functions to support or restrain γδ T cell expansion and effector function. One first-line IBD treatment, vedolizumab, inhibits T cell trafficking to the gut by blocking α_4_β_7_ integrin.^180^^,^^181^ While this approach would prevent peripheral γδ T cell infiltration, the depletion of tissue-resident cells remains a key limitation in restoring mucosal homeostasis. We propose that amplification or exploitation of the protective features of γδ T cells may prove a valuable therapeutic strategy, and it is possible that further insight into the crosstalk between γδ T cells and commensal bacteria may pave the way for novel microbiome-based therapies for IBD.
